# Revealing the cluster of slow transients behind a large slow slip event

**DOI:** 10.1126/sciadv.aat0661

**Published:** 2018-05-30

**Authors:** William B. Frank, Baptiste Rousset, Cécile Lasserre, Michel Campillo

**Affiliations:** 1 Department of Earth, Atmospheric and Planetary Sciences, Massachusetts Institute of Technology, Cambridge, MA 02139–4307, USA.; 2 Department of Earth and Planetary Sciences, University of California, Berkeley, Berkeley, CA 94720–4767, USA.; 3 Université de Lyon, UCBL, ENSL, CNRS, LGL-TPE, Villeurbanne, France.; 4 Institut des Sciences de la Terre, Université Grenoble Alpes, CNRS, IRD, Saint-Martin-d’Hères, France.

## Abstract

Capable of reaching similar magnitudes to large megathrust earthquakes [*M*_w_ (moment magnitude) > 7], slow slip events play a major role in accommodating tectonic motion on plate boundaries through predominantly aseismic rupture. We demonstrate here that large slow slip events are a cluster of short-duration slow transients. Using a dense catalog of low-frequency earthquakes as a guide, we investigate the *M*_w_ 7.5 slow slip event that occurred in 2006 along the subduction interface 40 km beneath Guerrero, Mexico. We show that while the long-period surface displacement, as recorded by Global Positioning System, suggests a 6-month duration, the motion in the direction of tectonic release only sporadically occurs over 55 days, and its surface signature is attenuated by rapid relocking of the plate interface. Our proposed description of slow slip as a cluster of slow transients forces us to re-evaluate our understanding of the physics and scaling of slow earthquakes.

## Teaser

A slow slip event is a cluster of small aseismic slip transients intermittently interrupted by relocking of the plate interface.

## INTRODUCTION

Slow slip events (SSEs) (*1*), like other slow earthquakes (*2*) such as tectonic tremor (*3*) and low-frequency earthquakes (LFEs) (*4*), occur downdip of the seismogenic zone where increasing temperatures and pressures transition the faulting style from brittle stick-slip to stable sliding (*5*–*7*). High pore fluid pressures maintained by the metamorphic dehydration of the downgoing slab impose small stress drops on any events that nucleate within this region and potentially inhibit fast rupture (*4*, *8*–*10*). Given that the seismic moment of tremors and LFEs is negligible with respect to the geodetic moment of slow earthquakes (*11*, *12*), SSEs are primarily observed with continuous Global Positioning System (GPS) measurements at the surface (*13*, *14*) whose temporal resolution is often limited to daily position solutions (*15*). Current numerical models constrained with these geodetic observations suggest that slow slip is the long-duration, steady rupture of the aseismic matrix on the subduction interface (*8*, *16*, *17*). The seismic asperities embedded within the aseismic fault material, which are responsible for tectonic tremor and LFEs, are transiently loaded by slow slip, resulting in accelerated seismicity rates (*18*). Recent studies have shown that it is possible to use these seismic crackles and pops to directly geodetically observe the underlying slow deformation (*19*, *20*).

**Fig. 1:**
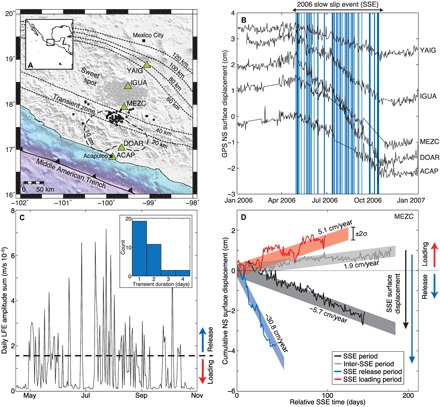
Breaking down a large SSE into its constituent slow transients. (**A**) Tectonic context of the subduction zone underneath Guerrero, Mexico. A large SSE in 2006 recorded by GPS (green triangles) accumulated more than 10 cm of slip (thick dashed contour) updip of LFE sources (black points, transient zone; gray points, sweet spot) (*22*). Depth contours of the subduction interface (*50*) are shown as thin dashed lines. (**B**) GPS displacement time series during 2006. The blue patches indicate the set of slow transients that exhibit tectonic release. (**C**) Daily LFE amplitude sums during the 2006 SSE. We identify slow transients on the days that the daily amplitude sum exceeds the established threshold (dashed black line). The insert shows the distribution of their durations. (**D**) Cumulative displacements at MEZC during the 2006 SSE before and after decomposition via the LFE amplitude sum in (C). The black trace represents the displacements during the 185-day slow slip duration (*16*) in (B), while the gray trace shows the inter-SSE displacements during the 185 days before the 2006 event. The red and blue traces respectively show the decomposed loading and release displacements (see main text). The shaded regions represent the estimated motion ±2σ during the slow slip duration of 185 days, of which there are no data at MEZC for 35 days. The slow slip–induced surface displacement of the cluster of slow transients during the release period of the 2006 SSE (blue arrow) is 40% larger than the surface displacement estimated from the GPS time series during the full slow slip duration (black arrow).

The subhorizontal subduction zone beneath Guerrero, Mexico shown in Fig. 1A hosts a slow slip cycle that releases more accumulated tectonic strain every 4 years than a moment magnitude (*M*_w_) 7 earthquake (*21*). Focusing on one of the most studied instances of this cycle, the continuous GPS displacement time series in Fig. 1B highlights a 6-month *M*_w_ 7.5 SSE in 2006. A geodetic kinematic model of this SSE reproduces the surface observations with a smooth rupture that lasts 185 days, accumulating more than 15 cm of slip on the plate interface (*16*). While the modeled slip history reproduces the long-period surface displacements as recorded by GPS, recent work has highlighted that there is coherent information at shorter time scales within the GPS time series that can be extracted using LFE/tremor activity as a guide (*20*). In this context, we perform a multidisciplinary investigation of the fine-scale evolution of the 2006 SSE using a dense catalog of LFEs (*22*).

## RESULTS

### Decomposition of surface motion via LFEs

Guerrero LFEs occur in two different source regions (see Fig. 1) (*22*). The sweet spot that is located furthest downdip exhibits a near-continuous stream of event bursts, with each burst thought to coincide with a small slip event (*23*). In the transient zone, closer to the trench within the main slow slip source region, there is a strong correlation between LFEs and geodetically observed SSEs (*18*). We therefore focus on the 34,389 LFEs spread over the 58 repeating sources in the transient zone to geodetically investigate the 2006 SSE.

We use this LFE catalog to decompose the surface displacement time series during the 2006 SSE (*16*) recorded at five GPS stations that lie between Acapulco and Mexico City. We first define the daily LFE activity in the transient zone as the product of the daily number of LFEs and the daily median LFE seismic amplitude, which we call the daily LFE amplitude sum time series (see Materials and Methods). Considering a high LFE activity in the transient zone as an indicator of when the subduction interface is slowly slipping (*19*, *20*), we then threshold the daily LFE amplitude sum time series in Fig. 1C to determine whether each daily GPS north-south (NS) displacement increment should represent tectonic release (slow slip) or loading (plate locking). Tectonic loading is represented by surface motion toward the north when the subducting Cocos plate is locked underneath the North American plate, while tectonic release corresponds to motion toward the south when the subduction interface decouples and built-up tectonic stress is released. By defining the daily LFE amplitude sum to include all transient zone LFE activity, we sacrifice spatial resolution to increase the temporal resolution of our analysis. This compromise allows us to geodetically detect slow slip on the same time scale as the sampling rate of the GPS time series.

### Intermittent and clustered evolution of slow slip

The decomposition shown in Fig. 1D of MEZC, the GPS station directly above the analyzed LFE activity and most sensitive to the slow slip in the vicinity of the transient zone, demonstrates that there are both loading and release regimes mixed together within the noisy surface displacements. We find not only a greater release displacement than the long-period signature that lasts three times longer but also northward loading during what was previously considered to be continuous slip. Once episodes of tectonic release are highlighted, visually striking periods of northward loading are evident in the GPS time series, such as the one in July 2006 (Fig. 1B). We observe this same separation of surface motion into two distinct regimes of release and loading on all of the analyzed GPS stations (see Materials and Methods and figs. S1 and S2). Considering continuous days of release as a single slow transient, we find that the average duration of slip is about 1 to 2 days (Fig. 1C). Taking into account the scattered temporal distribution of tectonic release over 55 days seen in Fig. 1B, this implies that slow transient slip is not continuous and is interrupted by intermittent locking of the plate interface. From the estimated loading velocity of 5.1 cm/year at MEZC that is only slightly lower than the plate convergence rate (6.4 cm/year), we infer that the subduction interface is at times completely locked during slow slip. This is also reported at multiple plate boundaries during the inter-SSE phase of the slow slip cycle (*20*), defined as the time period between large SSEs. This work shows that long-term loading rates are biased by intermittent release and locking that reveals strong plate coupling over short time scales.

**Fig. 2:**
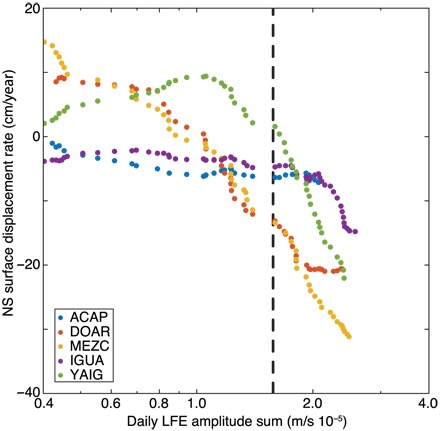
Estimating surface displacement rates via LFE amplitudes on the subduction interface. Surface displacement rates are computed at each GPS station as a function of increasing daily LFE amplitude sums. Southward surface motion in the direction of tectonic release, which is proportional to the motion on the decoupled interface at depth, becomes pronounced at LFE amplitudes greater than the established threshold (dashed line).

To home in on the fine-scale behavior of the slowly slipping plate interface, we compute the cumulative displacements at each GPS station as a function of increasing daily LFE amplitude sums, regardless of the tectonic regime. The smoothed slope of these cumulative displacements highlights the strong dependence of the surface displacement rate on LFE activity, as shown in Fig. 2. Because the surface displacement rate is proportional to the slip rate on the subhorizontal plate interface in Guerrero, we suggest that the evolution of the slip rate during slow slip mirrors the observed complex time history of the low-frequency seismicity (Fig. 1C). This is in contrast to previous theoretical (*8*, *17*) and data-driven (*16*) models of smooth large-scale SSEs and suggests that the complex time history of slow slip drives the intricate patterns of slow seismicity that are reported (*24*–*28*).

**Fig. 3:**
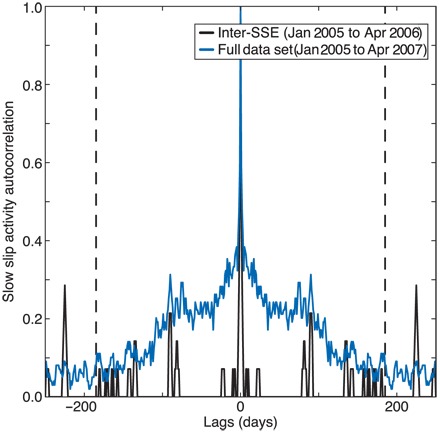
Slow slip as a cluster of slow transients. The autocorrelation of the slow slip activity time series in blue (see main text) indicates a temporally clustered distribution of slow transients with a falloff of 185 days (dashed lines), the duration of the 2006 SSE (*16*). The inter-SSE time period before the 2006 SSE (black) exhibits a Dirac at zero lag, indicative of a random occurrence of slow transients.

The intermittent slow deformation observed here is reminiscent of reports of temporally clustered LFE activity (*26*, *29*). To evaluate whether the timing of the slow transients mimics the clustered distribution of LFE activity, we analyze the 2 1/2-year duration of the LFE catalog from 1 January 2005 to 15 April 2007. We first divide the previous LFE amplitude sum threshold by two to account for the lower LFE rates before and after the 2006 event (*18*). We then generate a regularly sampled binary slow slip activity time series: 1 on days when the daily LFE amplitude sum exceeds the threshold and slow slip is considered to occur, and 0 for every other day. The autocorrelation of this time series in Fig. 3 shows a smooth falloff from zero lag that indicates that the timing of the slow transients is not random and their occurrence is clustered (*26*). We also observe that the clustering falls off until 185 days, which corresponds to the long-period duration of the 2006 SSE. This demonstrates that the observed intermittent tectonic release during the large SSE represents a clustered occurrence of slow transients, where each slow transient triggers and is triggered by other slip events. If we analyze only the time period before the 2006 SSE, we see that this clustering disappears and is replaced by a delta function at zero lag, implying a random temporal distribution of slow slip before the large 2006 event (*26*).

## DISCUSSION

### Redefining slow slip as a cluster of slow transients

A new description of slow slip emerges from the set of observations presented here: Once the subduction interface decouples, it provokes a cluster of short-duration slow transients that can last for several months. Comparing the average slow transient duration of 1 to 2 days that we observe to the 6-month long-period signal (*16*) in Fig. 1, we suggest that each slow transient is a short-duration slip pulse. Where the conditions are right for tremors and LFEs (*4*, *9*), each individual slip pulse briefly increases the stressing rate on nearby asperities, triggering a burst of seismic activity (*26*) before the subduction interface relocks. Surface geodetic observations at long time scales are unable to capture this fine-scale activity and only sample the smooth envelope of a cluster of slow transients. Our conceptual model thus accounts for both the aseismic and seismic observables of large SSEs. This description of a large SSE is also consistent with observations earlier in the slow slip cycle, where intermittent release and locking define the inter-SSE period in between SSEs (*20*).

To produce the observed highly variable slip rate and clustering behavior, we suggest that the frictional heterogeneity of the subduction interface is the dominant factor in controlling the evolution of slow slip (*30*). This implies that the heterogeneity that governs the ruptures of megathrust earthquakes in the seismogenic zone (*31*, *32*) is preserved during subduction and plays a major role in how tectonic motion is accommodated at greater depths. Previous numerical works (*33*–*37*) have also suggested that the frictional heterogeneity along a fault can reproduce many of the observables of slow earthquakes, with a complex evolution of slip on brittle asperities controlled by slow aseismic slip in the surrounding fault material. Constant background aseismic slip as the driving mechanism behind slow slip is not consistent, however, with the significant periods of tectonic loading that we observe during slow slip (Fig. 1). Our results negate the possibility of such a large-scale slow aseismic slip front that would link the individual slow transients into a cluster because the observed loading rates imply a locked plate interface. Any potential mechanism behind the clustered slow transients we observe here would have to be able to govern the interaction between slow transients along a locked fault. One such mechanism for which there is abundant geological evidence (*38*–*41*) is the rapid diffusion of high pore fluid pressures during faulting at depth (*18*, *42*, *43*).

Another significant consequence of the intermittent relocking we observe during slow slip is the attenuation of the surface motion, as recorded by GPS. This accounts for the 40% larger surface displacements that we observe during the release regime in Fig. 1D compared to the long-period geodetic estimates (*16*). We consequently infer that the long-period measurements of surface displacement that inform previous models of large SSEs systematically underestimate their moment magnitude. We note that there is the possibility that this bias could also affect previously determined source locations of large SSEs. This intermittent locking likely depends on the dominant style of faulting that varies with depth (*5*, *6*), implying a moment underestimation that varies along the plate interface with distance from the trench. This would affect the distribution of surface displacements during slow slip that inform geodetic fault slip inversions, consequently affecting the inferred source location of slow slip.

## CONCLUSIONS

By breaking down a large SSE into a cluster of slow transients, we demonstrate that previous studies of large SSEs both overestimate their duration *T* and underestimate their moment magnitude *M*. Our multidisciplinary analysis of the 2006 SSE yields a three times shorter duration and, assuming the same spatial distribution of slip as a previous study (*16*), a moment that is at least 40% larger than the previous geodetic estimate. If we impose a similarly shorter duration and larger moment on all large SSEs observed at plate boundaries, the proposed *M* ~ *T* slow earthquake scaling (*44*) will not shift directly to a classical *M* ~ *T*^3^ earthquake scaling; it will instead likely satisfy a scaling relationship with an exponent between 1 and 2 that is consistent with fractal distributions of fault slip (*45*–*47*). However, this bias we observe will displace all of the observations of large SSEs that constrain the proposed *M* ~ *T* slow earthquake scaling (*44*) at long durations and large moments. Another possible interpretation is that each of the short-duration slow transients should be characterized as separate slow earthquakes. However, this ignores the characteristic clustering signature (*26*) that links temporally disparate slow transients together to create a large SSE. In any case, our results contribute to a growing body of evidence (*48*, *49*) that we must re-evaluate our understanding of the physics and scaling of slow earthquakes in light of new observational constraints.

## MATERIALS AND METHODS

### Daily LFE amplitude sums

We computed the daily amplitude sum of LFEs as follows

$$\bar{A}(t)=N(t)A(t)$$ (1)

where *t* is time (in days), *N*(*t*) is the daily count of LFEs, and *A*(*t*) is the daily median amplitude of the cataloged LFEs (*22*). The daily median LFE amplitude *A*(*t*) is determined as

$$A(t)=\underset{i}{\text{median}}[\underset{s}{\text{median}}(\frac{\sum_{c}^{C}a_{i}^{s,c}}{C})]$$ (2)

where *a*_*i*_ represents the peak amplitude of the *i*th LFE on a given day *t*, *s* represents the 10 seismic stations used to generate the LFE catalog, and *c* represents the *C* = 3 components.

### Decomposition of GPS displacement increments via LFE amplitude sums

We first computed the daily GPS NS displacement increments Δ*x*_*s*_ at each station *s* as

$$\Delta x_{s}(t)=x_{s}(t+1)-x_{s}(t)$$ (3)

where *t* is time in days and *x*(*t*) is the GPS position time series. We propagated the observational position errors ϵ_*s*_(*t*) to the displacement increment errors Δϵ_*s*_(*t*) as follows

$$\Delta \epsilon_{s}(t)=\sqrt{\epsilon_{s}{\left(t\right)}^{2}+\epsilon_{s}{\left(t+1\right)}^{2}}$$ (4)

We then analyzed the daily LFE amplitude sums with respect to some threshold to determine when the subduction interface was slipping and releasing tectonic stress or loading and accumulating stress. For example, during tectonic release, there should be significant LFE activity associated with slow slip and a consequent high-amplitude sum greater than the established threshold. During the loading regime, there should be little to no activity and a low-amplitude sum smaller than the threshold while the plate interface is locked and coupled.

After this sorting was completed, we had four different sets of displacement increments that were each associated with a different tectonic regime: the long-period 185-day SSE duration; the inter-SSE period, defined here as the 185 days before the 2006 SSE; and the release and loading regimes during the SSE as defined by LFE activity. Because we observed a stationary distribution of displacement increments for each regime, we estimate the displacement uncertainty as the mean of the observational errors Δϵ_*s*_(*t*) for each regime. We report this average observational error ${\bar{\Delta \epsilon }}_{s}(t)$ multiplied by four as the 4σ width of the shaded regions in Fig. 1 and fig. S1. We know that the mean was not a stable estimate of the average GPS displacement velocity because only a fraction of the displacement increments contributed to the estimate (*20*). We therefore determined the average surface velocity as the slope of the linear regression of the cumulative displacement time series as it took into account every displacement increment datum. Given that the displacement increments represent a relative displacement that occurs in a given regime regardless of its time stamp, the measured velocity from the cumulative displacement time series of a random resampling of the displacement increments should be the same. We therefore randomly resampled the displacement increments (allowing for a given datum to be selected multiple times) 10,000 times for each regime and computed a cumulative displacement time series for each resampling. We then performed a linear regression of all 10,000 iterations, with each point weighted by ${\bar{\Delta \epsilon }}_{s}{\left(t\right)}^{-2}$, to determine the average velocity for each regime as the slope of the best-fit linear trend. We then computed the surface displacement as the average velocity multiplied by the duration of that regime, as defined by the daily LFE amplitude sum time series in Fig. 1C. In such a way, we avoided any biases associated with data gaps in the geodetic time series. As described above, we then reported the uncertainty of the displacement as the average observational error.

Now that we could compute displacements and velocities for each regime given some threshold, we tested all possible thresholds to determine the best threshold that yielded the largest different displacement, defined as the loading displacement minus the release displacement. Given that there are a finite number of daily LFE amplitude sums, we used each as a potential threshold and performed the GPS decomposition for the loading and release regimes, as described above. We then stacked the differential displacements over the five analyzed GPS stations in fig. S3 and picked the threshold that produced the maximum stacked differential displacement.

### Robustness of GPS displacement decomposition

The network sum of the estimated release displacements provided a single quantity for each iteration that represented how effective the decomposition into loading and release was. To evaluate whether the observed decomposed displacements in Fig. 1 and fig. S1 could happen by chance, we used the following bootstrap analysis.

We randomly shuffled the time *t* of the daily LFE amplitude sum time series without modifying the time *t* of the GPS displacement increments and reperformed the decomposition at all stations. We performed this shuffling 10,000 times and compared the observed network release displacement to the distribution of random network release displacements in fig. S2. Given that the observed displacement is more than 3σ from the mean of the random network release displacements, we concluded that there was a negligible chance that our observations resulted from a random decomposition.

## Supplementary Material

http://advances.sciencemag.org/cgi/content/full/4/5/eaat0661/DC1

## SUPPLEMENTARY MATERIALS

Supplementary material for this article is available at http://advances.sciencemag.org/cgi/content/full/4/5/eaat0661/DC1 fig. S1. Decomposition of surface displacement increments into loading and release in Guerrero, Mexico. fig. S2. Comparing observed network sum of release displacement (dashed blue line) to a random shuffling of the daily LFE amplitude sum time series. fig. S3. Determining the LFE amplitude sum threshold.
