# Supplementary material for: Revealing the cluster of slow transients behind a large slow slip event
Source: Sci Adv. 2018 May 30;4(5):eaat0661. doi: 10.1126/sciadv.aat0661 (PMC5976274; doi:10.1126/sciadv.aat0661)
Supplement: http://advances.sciencemag.org/cgi/content/full/4/5/eaat0661/DC1 [file supp_4_5_eaat0661__index.html]

Science Advances | Science Advances

## Supplementary Materials

**This PDF file includes:**

- fig. S1. Decomposition of surface displacement increments into loading and release in Guerrero, Mexico.
- fig. S2. Comparing observed network sum of release displacement (dashed blue line) to a random shuffling of the daily LFE amplitude sum time series.
- fig. S3. Determining the LFE amplitude sum threshold.

Download PDF

**Files in this Data Supplement:**

- Adobe PDF - aat0661\_SM.pdf
