# Supplementary material for: Revealing the cluster of slow transients behind a large slow slip event
Source: Sci Adv. 2018 May 30;4(5):eaat0661. doi: 10.1126/sciadv.aat0661 (PMC5976274; doi:10.1126/sciadv.aat0661)
Supplement: http://advances.sciencemag.org/cgi/content/full/4/5/eaat0661/DC1 [file aat0661_SM.pdf]

Science Advances

AAAS

advances.sciencemag.org/cgi/content/full/4/5/eaat0661/DC1

Supplementary Materials for

Revealing the cluster of slow transients behind a large slow slip event

William B. Frank, Baptiste Rousset, Cécile Lasserre, Michel Campillo

Published 30 May 2018, *Sci. Adv.* **4**, eaat0661 (2018)
DOI: 10.1126/sciadv.aat0661

This PDF file includes:

- fig. S1. Decomposition of surface displacement increments into loading and release in Guerrero, Mexico.
- fig. S2. Comparing observed network sum of release displacement (dashed blue line) to a random shuffling of the daily LFE amplitude sum time series.
- fig. S3. Determining the LFE amplitude sum threshold.

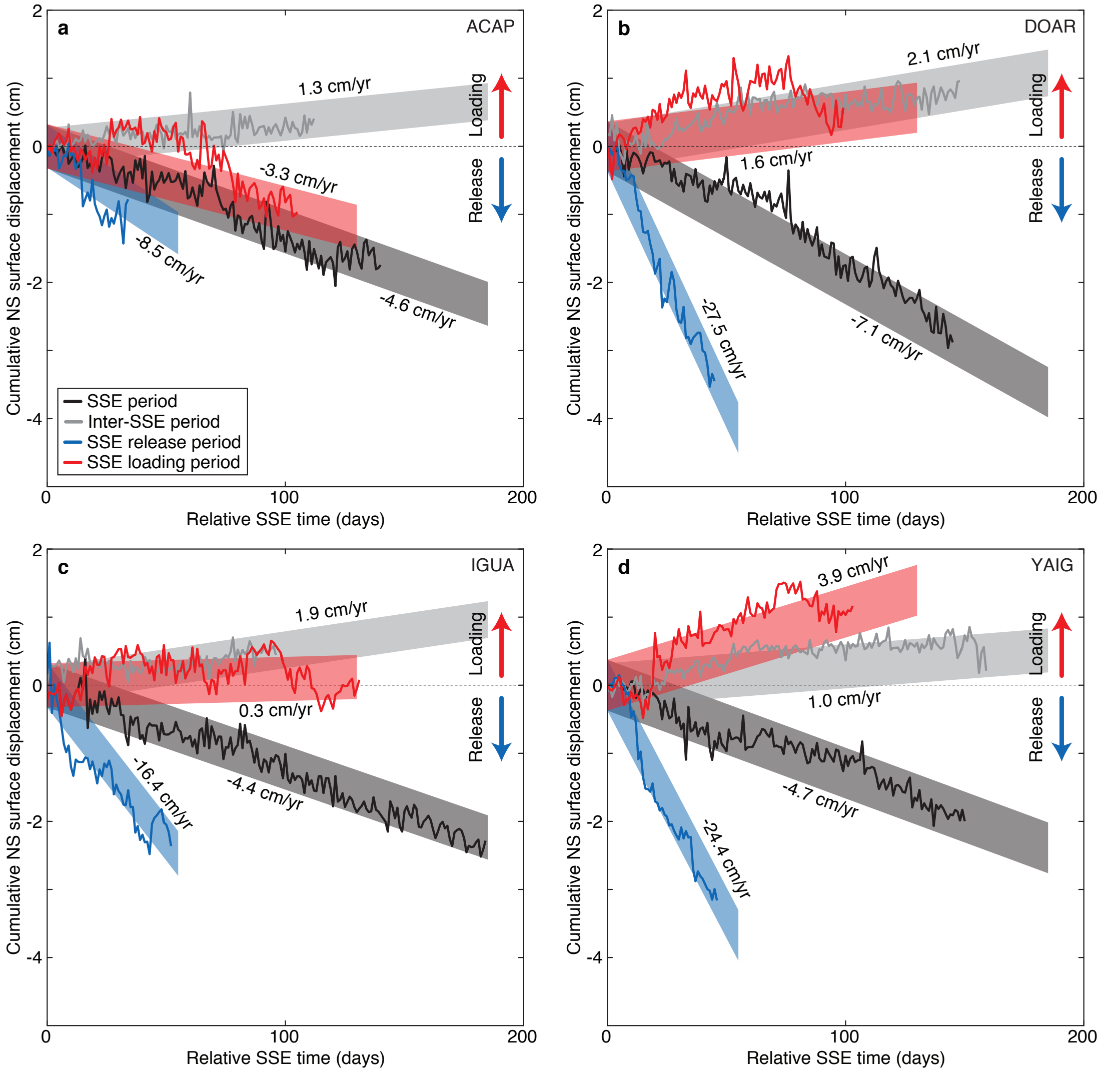

fig. S1. **Decomposition of surface displacement increments into loading and release in Guerrero, Mexico.** The black trace in each panel is the full slow slip duration (185 days) GPS time series, while the gray trace shows the inter-SSE (Slow Slip Event) motion during the 185 days prior to the 2006 slow slip event. The red and blue traces respectively indicate the cumulative loading and release displacements at ACAP (a), DOAR (b), IGUA (c), and YAIG (d) during the 2006 slow slip event after decomposition (see text). The shaded regions represent the estimated motion $\pm 2\sigma$ during the full slow slip duration taking into account data gaps at each station. The decomposition at all stations successfully separates release and loading regimes within the GPS time series: displacement during release trends faster southwards, and displacement during loading trends faster northwards, compared to the GPS time series during the full slow slip duration.

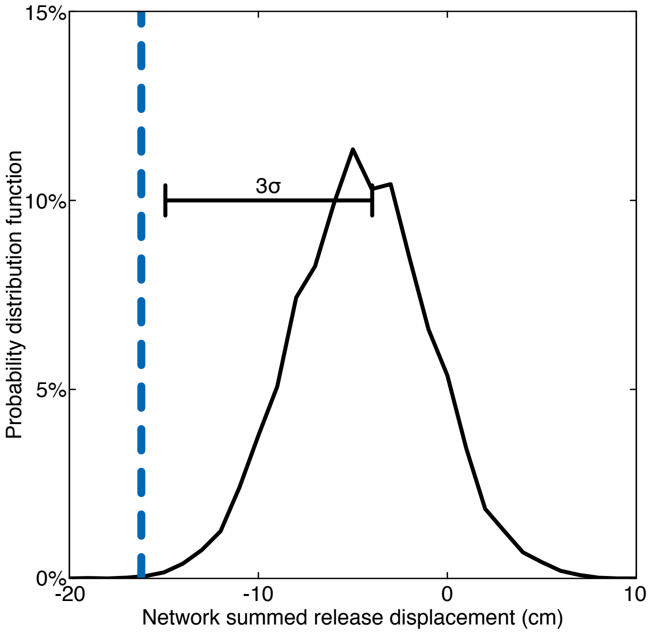

fig. S2. **Comparing observed network sum of release displacements (dashed blue line) to a random shuffling of the daily LFE amplitude sum time series.** The daily amplitude sums from Fig. 1c are randomized before computing the GPS decomposition into loading and release. This is performed 10,000 times and the resulting distribution of network summed release displacements are plotted in black.

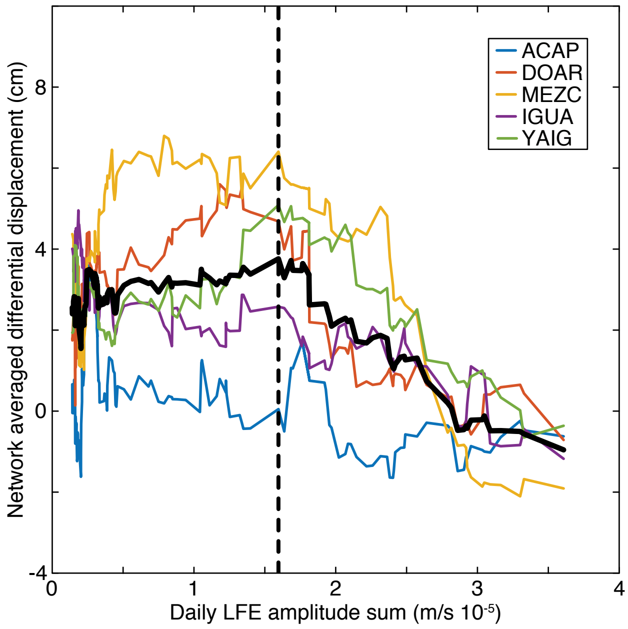

fig. S3. **Determining the LFE amplitude sum threshold.**
The differential displacement (loading displacement to the North minus release displacement to the South) is computed as a function of potential daily LFE amplitude sum thresholds for each station (colored lines). After computing the network average of these differential displacements (black line), we then choose the threshold that produces the maximum averaged differential displacement (dashed black line).
